# Energy Reduction Effect of the South-to-North Water Diversion Project in China

**DOI:** 10.1038/s41598-017-16157-z

**Published:** 2017-11-21

**Authors:** Yong Zhao, Yongnan Zhu, Zhaohui Lin, Jianhua Wang, Guohua He, Haihong Li, Lei Li, Hao Wang, Shan Jiang, Fan He, Jiaqi Zhai, Lizhen Wang, Qingming Wang

**Affiliations:** 1 State Key Laboratory of Simulation and Regulation of Water Cycle in River Basin, China Institute of Water Resources and Hydropower Research, Beijing, 100038 China; 2 International Center for Climate and Environment Sciences (ICCES). Institute of Atmospheric Physics, Chinese Academy of Sciences, Beijing, 100029 China; 3 Collaborative Innovation Center on Forecast and Evaluation of Meteorological Disasters, Nanjing University of Information Science and Technology, Nanjing, 210044 China; 4 State Nuclear Electric Power Planning Design and Research Institute, Beijing, 100094 China

## Abstract

The North China Plain, with a population of approximately 150 million, is facing severe water scarcity. The over-exploitation of groundwater in the region, with accumulation amounts reaching more than 150 billion m^3^, causes a series of hydrological and geological problems together with the consumption of a significant amount of energy. Here, we highlight the energy and greenhouse gas-related environmental co-benefits of the South-to-North Water Diversion Project (SNWDP). Moreover, we evaluate the energy-saving effect of SNWDP on groundwater exploitation based on the groundwater-exploitation reduction program implemented by the Chinese government. Our results show that the transferred water will replace about 2.97 billion m^3^ of exploited groundwater in the water reception area by 2020 and hence reduce energy consumption by 931 million kWh. Further, by 2030, 6.44 billion m^3^ of groundwater, which accounts for 27% of the current groundwater withdrawal, will save approximately 7% of Beijing’s current thermal power generation output.

## Introduction

As a result of the mismatch between social activities and water resources, the North China Plain (hereafter, NCP) has experienced increasing water-scarcity problems during the past half century^1–3^, which is a particularly serious concern in Beijing, Tianjin, Hebei, and Henan Provinces^4^. To meet requirements for regional socio-economic development, the underground water in this region has been over-exploited for a long period^5,6^, with the number of wells increasing from 1,800 in the 1960s to 15 million in 2011^7,8^. The long-term groundwater monitoring results show that the average shallow groundwater level based on the depth of the water table to the ground in Hebei Province has decreased from 7.23 m in 1983 to 11.52 m in 1993^7^, and the maximum depth the shallow phreatic groundwater level in Hebei Province can reach is 40 m. Similarly, the water table in the deep confined aquifer is also decreasing. A number of large cones of depression were formed^9^, and the water table in the cone center dropped from 50 m in the 1980s to 70 m in 2013 in central and southeastern Hebei.

Moreover, groundwater over-exploitation has caused problems such as river dry-up, lake shrinkage, aquifer depletion, and land subsidence in many areas^10,11^. Meanwhile, the energy used for water pumping represents an assignable energy use in the water sector^12,13^. Therefore, it is important to exploit the regional water-energy nexus and highlight the energy and greenhouse gas-related environmental co-benefits of water resources management^14^.

**Figure 1:**
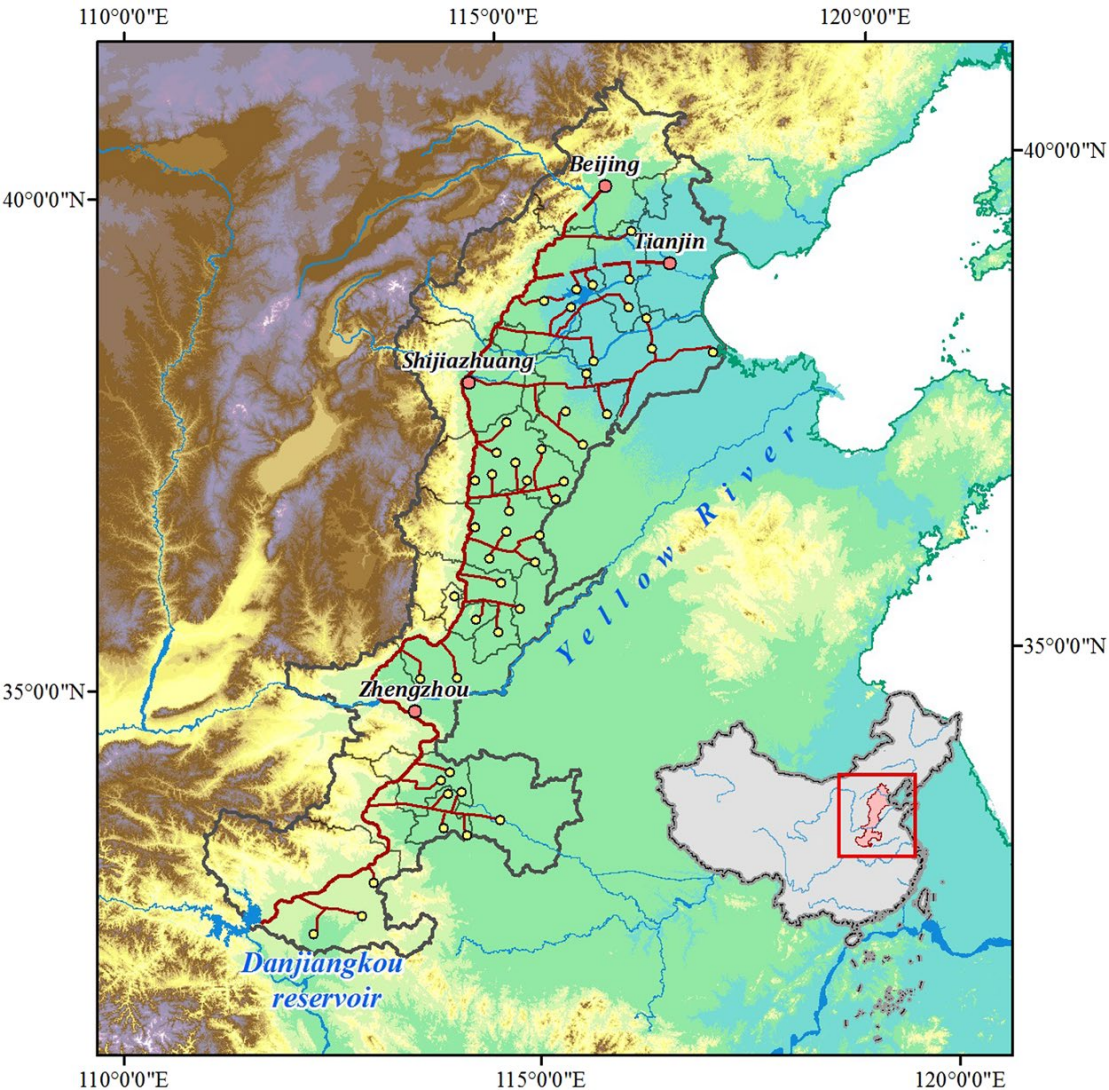
Middle route of South-to-North Water Diversion Project. The project transfers water from the Danjiangkou Reservoir on the upper reaches of the Hanjiang River, which is the largest branch of the Yangtze River, to NCP, where there is a shortage of surface water. Annually diverted water is expected to be 9.5 billion m^3^ and will resolve industrial and domestic water shortages in more than 20 large and medium-sized cities along the route in Henan and Hebei Provinces, Tianjin Municipality, and Beijing Municipality. The maps were created using ArcGIS 10.2 (http://www.esri.com/software/arcgis/arcgis-for-desktop).

In recent decades, total water consumption in the NCP area has shown a gradual increasing trend^9^, and a large amount of energy has been consumed by the water-pumping system^12,13^. In the southern Haihe River Plain, which accounts for 30% of the NCP area, the energy used to pump shallow groundwater for irrigation can reach 1.63 billion kWh^15^. With the implementation of the middle route of the South-to-North Water Diversion Project (SNWDP; Fig. 1), the regional water supply structure has changed gradually, which will reduce groundwater exploitation in the area^16,17^.

The aim of this study is to quantify the future changes in groundwater pumping and the resulting energy consumption changes in the middle route of the SNWDP area. For this purpose, we analyze the current spatial distribution of the groundwater exploitation and further estimate the related energy consumption using the groundwater level and exploitation well information. Then, the reduced amount of groundwater exploitation in 2020 and 2030 will be calculated based on the scenario analysis of the total volume quota of groundwater replacement in the SNWDP area. Moreover, the reduced energy consumption from reduced water pumping in 2020 and 2030 will be estimated in addition to the influence of the middle route SNWDP on carbon emissions.

With consideration of data availability, the 10-year period from 2004 to 2013 is the reference period for comparison, which is just before the official operation of the middle route of the SNWDP.

## Results

### Spatial distribution of groundwater energy consumption from 2004 to 2013

The NCP has relied on groundwater resources heavily in the past. Despite the increasing use of reclaimed water and desalinated seawater year after year, groundwater is still the main source of water supply in the region, although its contribution to the total water withdrawal varies in different parts of the NCP. On average, 23.77 billion m^3^ of groundwater is extracted annually^9^, which accounts for 69% of total water withdrawal.

**Figure 2:**
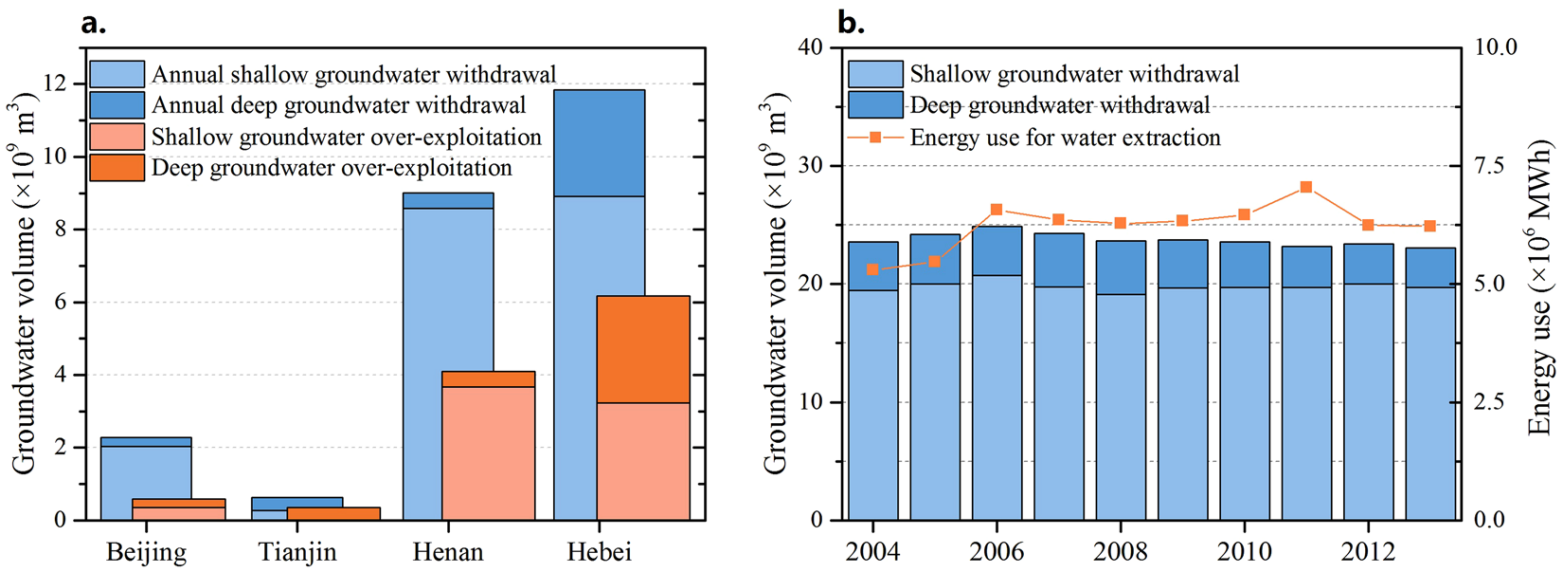
Volume of groundwater withdrawal and energy use for groundwater of the South-to-North Water Diversion Project area. Figure 2(a) shows the 10-year volumes of the average annual extraction of shallow groundwater (light blue) and deep groundwater (dark blue) compared with the over-exploitation volume of shallow and deep groundwater (light and dark orange, respectively). Figure 2(b) shows the annual groundwater withdrawal from 2004 to 2013 and the related energy consumption.

With the large water demand for urban development and agriculture, the use of regional groundwater has far exceeded the amount of recharge into the aquifer. The average annual groundwater overdraft is 11.21 billionm^3^ in the NCP, and about 35% of that is from deep groundwater. From Fig. 2a, we find that the annual groundwater exploitation in Beijing, Tianjin, Hebei, and Henan amounts to 2.28, 0.63, 11.85, and 9.01 billion m^3^, respectively. Meanwhile, a larger amount of withdrawn shallow groundwater was found in Beijing and Henan, while in Hebei, both the shallow and deep groundwater are severely over-exploited. Hebei suffers from the most severe groundwater over-exploitation. During the last 10 years, the maximum depth of the shallow groundwater depression area decreased from 52 m to 70 m, and the water table in the deep groundwater cone center dropped to 100 m below surface in 2013 in southeastern Hebei.

Due to the large amount of groundwater withdrawal, a significant amount of energy is consumed by groundwater lifting, which is dependent on the amount of groundwater mining, groundwater level, and pumping efficiency^18^. The annual groundwater energy consumption from 2004 to 2013 is thus calculated and shown in Fig. 2b. Even though the total amount of groundwater withdrawal was slightly reduced from 2008, the related energy consumption increased gradually from 5.3 billion kWh in 2004 to 6.3 billion kWh in 2013, which can be ascribed to the continued decline of groundwater levels.

**Figure 3:**
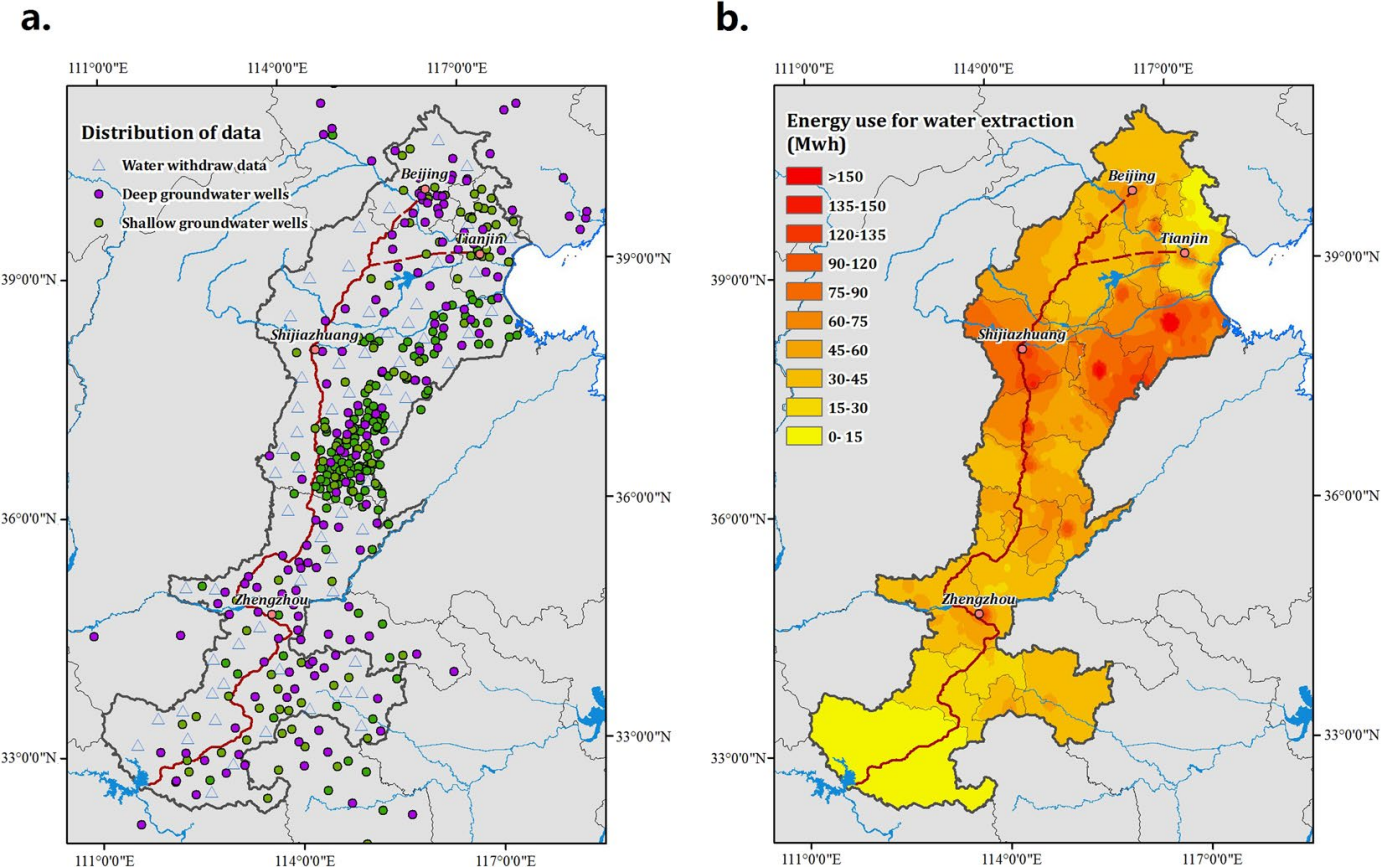
Annual average energy consumption of groundwater exploitation. Figure 3(a) shows the distribution of the water withdrawal data and the groundwater data that are used in this study, and Fig. 3(b) shows the annual average energy use of per unit-area groundwater exploitation. The energy use for lifting water is more concentrated in large cities and the irrigated area in Hebei Province. The maps were created using ArcGIS 10.2 (http://www.esri.com/software/arcgis/arcgis-for-desktop).

The spatial distribution of groundwater pumping energy consumption is shown in Fig. 3. As an important source of urban and industrial water, groundwater mining is concentrated around cities and counties. The annual average energy consumption from 2004 to 2013 in Beijing, Tianjin, Hebei, and Henan is 0.54, 0.20, 3.67, and 1.94 billion kWh/year, respectively. Hebei is an important granary of China in the NCP^19^, agriculture also relies on groundwater, and approximately 8.90 billion m^3^ of groundwater has been exploited annually to secure food production, which represents about 75% of Hebei’s total groundwater withdrawal. With the double influence of the groundwater level and exploitation volume, the energy consumption in Northeast Hebei Province is significant.

### Prediction of groundwater reduction in the future and energy consumption

On December 12, 2014, operation of the middle route of the SNWDP was officially initiated^16^, flowing through the eastern plain of North China. By the end of 2016, the SNWDP had transferred 5.85 billion m^3^ water to Beijing, Tianjin, Hebei, and Henan Provinces within two years. After the completion of its supporting facilities, the middle route of the SNWDP is expected to deliver 9.5 billion m^3^ of water to NCP each year. Considering the water loss by evaporation, the net available transferred water is approximately 8.52 billion m^3^ ^20^, which is equivalent to 36% of current groundwater withdrawal. With an approximate 100-m elevation difference between the Danjiangkou Reservoir and Beijing, the diverted water can simply flow under the influence of gravity with little dependence on pumping stations^21^. Therefore, the water diversion project will greatly reduce groundwater exploitation and contribute to regional energy saving.

At present, the groundwater exploitation reduction program has been initiated by the Chinese government^20^. According to the water resources planning for the water reception area of the SNWDP, the water allocation principles are strictly created for each sector and city. Moreover, the total volume quota of groundwater exploitation from 2015 to 2020 has been established by the provincial, city, and county-level administrators. This reduction program aims to reduce the use of groundwater for cities and industries by 2020, including 1.65 billion m^3^/year of shallow groundwater exploitation and 1.32 billion m^3^/year of deep groundwater exploitation, which is equivalent to 60% of the current groundwater over-exploitation in the area.

The long-term, groundwater management targets have also been established based on the balance of the groundwater between extraction and replenishment^20^. In addition to urban and industrial groundwater extraction control, the water sources in rural areas will also be basically adjusted by replacing the over-exploitation of groundwater with the water diversion of the SNWDP. That is, 3.39 and 3.05 billion m^3^ per year of shallow and deep groundwater extraction, respectively, will be reduced in the reception area. Additionally, if a second phase of the middle route of the SNWDP is identified, groundwater exploitation will be further reduced^20^.

The predicted reductions in groundwater use of the water reception areas based on government planning are shown in Table 1. By 2020, the SNWDP will mainly replace the over-exploitation volume in the urban and key industrial areas in the NCP, which is equivalent to 12.5% of current groundwater withdrawal. The energy consumption of groundwater exploitation in the future is estimated using the spatial distribution of the latest 10-year average groundwater table and the groundwater reduction data planned by the city or county-level administration and applying the formula for groundwater energy consumption^18^. In 2016, the SNWDP water was already linked with the urban water supply system in Beijing, Tianjin, and Henan Provinces. Therefore, after the completion of the water supply network in Hebei Province, the over-exploitation situation in the urban area in the NCP will be gradually controlled.

**Table 1:** Predicted reduction in groundwater withdrawal of the middle route of the South-to-North Water Diversion Project area.

Area	Current annual ground water extraction	Planned net water transfer volume	Predicted reduction in groundwater withdrawal around 2020		Predicted reduction in groundwater withdrawal around 2030	
			*Shallow groundwater*	*Deep groundwater*	*Shallow groundwater*	*Deep groundwater*
Beijing	2.28	1.05	0.40	0.24	0.4	0.24
Tianjin	0.63	0.85	0.00	0.26	0.00	0.35
Hebei	11.85	3.04	1.08	0.72	1.00	2.04
Henan	9.01	3.58	0.17	0.1	1.99	0.42
**Total**	**23.77**	**8.52**	**1.65**	**1.32**	**3.39**	**3.05**

Notes: unit = billion m^3^.

By the 2020 s, Hebei Province is planning to reduce 1.8 billion m^3^ of groundwater in total, which is 65% and 54% of the total reduction volume of shallow and deep groundwater, respectively. The annual average shallow groundwater table in Hebei is 16 m, but, in the depression zone, the water table is over 70 m. The average deep groundwater table in the northeast part of Hebei reaches 100 m. If the fluctuation of the groundwater level is not taken into consideration, energy consumption will be reduced by 591 million kWh by 2020.

**Figure 4:**
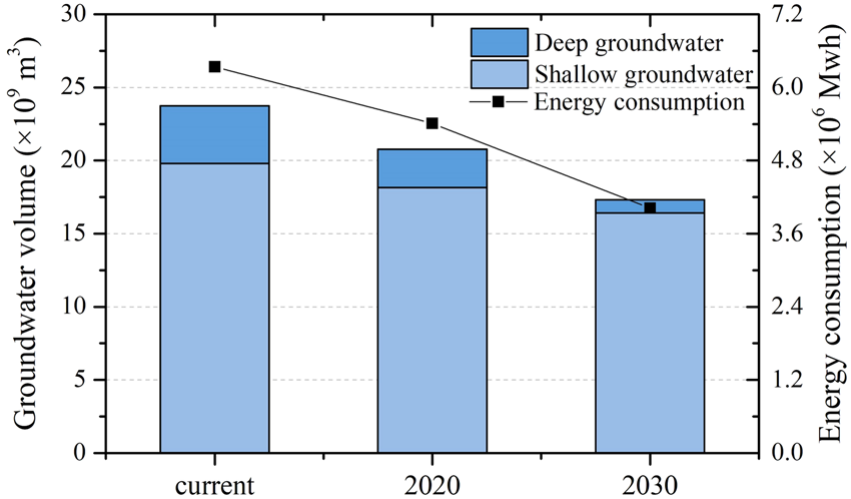
Future groundwater consumption and related energy consumption. In the South-to-North Water Diversion Project area, the average annual groundwater extraction from 2004 to 2013 was 23.77 billion m^3^ and the energy consumption of groundwater exploitation was 6.35 billion kWh. The predicted groundwater withdrawals in 2020 and 2030 are 20.79 and 17.32 billion m^3^, and predicted energy consumption is 5.41 and 4.0 billion kWh, respectively.

The predicted reductions in total groundwater withdrawal around 2020 in Beijing, Tianjin, and Henan Provinces are expected to be 0.64, 0.26, and 0.27 billion m^3^, respectively. The NCP regional energy savings in 2020 are expected to be 931 million kWh (Fig. 4).

Further, the groundwater management in rural areas will be gradually put into effect after 2020. NCP is expected to gradually achieve groundwater replenishment balance by 2030. Most of the over-exploited volume of shallow and deep groundwater will be replaced by SNWDP representing 27% of current groundwater withdrawal. The energy consumption will be reduced by 2.32 billion kWh. That is equivalent to 7% of Beijing’s current thermal power generation output (33.56 billion kWh^22^). China’s energy consumption is dominated by coal^23^. The decrease in groundwater exploitation is the equivalent of a reduction of 244 and 607 k tons of carbon dioxide emissions in 2020 and 2030, respectively. Regional air pollution is expected to be substantially reduced.

### Uncertainty analysis

The consistency of the SNWDP project is an issue to which many experts and scholars pay close attention^24^. It also has a profound influence on socio-economic development in the NCP area and brings great uncertainty to the groundwater replacement effect. Here, we focus on the uncertainty of the water source project and the uncertainty of water demand in the NCP area.

The uncertainty of the water source project includes the changes in water projects for both the Danjiangkou Reservoir on the Han River and the prospective new water source project in the NCP area. The deliverable water quantity from the Danjiangkou reservoir is limited by its capacity size and also influenced by regional climate change, the water needs of the downstream areas, and national policy adjustment. The local water resources in the NCP are limited; therefore, the new water supply will be mainly from desalination or recycled water plants. In addition, water demand in the NCP is also a key subject that needs to be discussed. The impacts of climate change and socio-economic development are major uncertainties that require quantitative analysis.

Four high-resolution general circulation models (GCMs; Table 2) are used to analyze the influence of climate change on water resources in the Danjiangkou Reservoir. Under the mid greenhouse gas emission scenario of representative concentration pathway (RCP) 4.5^25^, the predicted average mean of water resources from 2020 to 2035 in the upper reaches of the Danjiangkou Reservoir is 32.72 ± 5.63 billion m^3^, which is 6% less than the average annual water resources from 1956 to 2000. The high greenhouse gas emission scenario of RCP 8.5^26^ shows that the Han River Basin will face a relatively dry period; the average water resources around 2030 in the upper reaches are 30.38 ± 5.20 billion m^3^, which is 14% less than current conditions.

**Table 2:** Analysis of water resource changes from 2020–2035.

No.	GCMs	Average annual transferable water from Danjiangkou reservoir		Average water resource in the NCP area	
		RCP 4.5	RCP 8.5	RCP 4.5	RCP 8.5
1	CanESM2^42^	14.2	12.4	28.3	30.4
2	CNRM-CM5^43^	9.4	4.0	24.3	19.5
3	GISS-E2-R^44^	10.8	7.7	27.4	21.3
4	MRI-CGCM^45^	14.7	13.3	20.9	26.8
5	**Ensemble** **Mean**	**12.3**	**9.3**	**25.2**	**24.5**

Source: Intergovernmental Panel on Climate Change, Fifth Assessment Report, models.

Notes: unit = billion m^3^.

**Figure 5:**
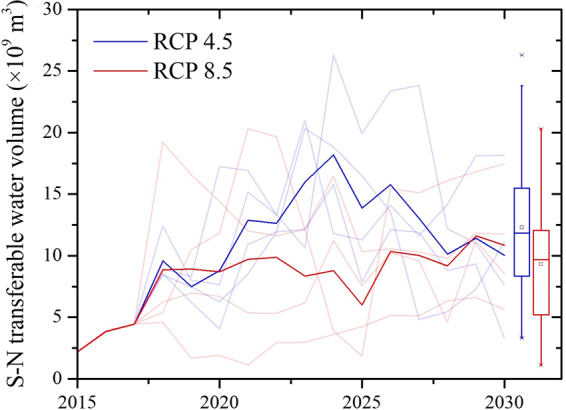
Future transferable water from the middle route of the South-to-North Water Diversion Project area. The ensemble mean of transferable water from the middle route of the South to North Water Diversion Project is estimated with four high-resolution GCMs using RCP 4.5 (blue) and 8.5 (red) climate change scenarios. The background lines show the estimated results from individual models.

The Hanjiang River Basin has natural advantages regarding industrial and agricultural development because of sufficient water and mineral resources. With economic development, the water withdrawal in Hanjiang River Basin will increase by 7% compared with 2013^27^, which is equivalent to 1.1 billion m^3^ water. As Fig. 5 shows, the average annual transferable water volume under the RCP 4.5 scenario is 12.29 ± 6.49 billion m^3^. Under the RCP 8.5 scenario, the Han River Basin will experience a dry period; the annual transferable water volume is 8.89 ± 5.33 billion m^3^, which is 2% less than the original water diversion plan. Considering the multi-year regulating ability of the Danjiangkou reservoir, rains from a high flow year will accumulate in the reservoir for use in a drier year, and thus we believe the impact can be ignored.

The climate will influence not only the amount of the water supply but also the water demand in the NCP. Based on the RCP simulation results from four GCMs, the precipitation in the NCP area shows the same trend as the water supply area in Hanjiang River Basin. Under the RCP 4.5 scenario, the average water resources around 2030 are 2% more than the current conditions, which are equivalent to 0.5 billion m^3^. Under the RCP 8.5 scenario, the average water resources are 24.5 billion m^3^, which is 1% less than the current situation. The results show that water resources in the NCP area may demonstrate little change. The simulation results for the individual climate model are shown in Table 2, and standard deviations under the RCP 4.5 and 8.5 scenarios are 2.9 and 4.3 billion m^3^, respectively.

Due to substantial environmental and resource pressures, population growth in Beijing and Tianjin has been strictly controlled in recent years^28^. Agricultural water withdrawal in the NCP area is also strongly controlled^29,30^. Creating a modern industry is the future development goal for China; thus, it is estimated that the industrial value-added of the NCP area will increase by 36.5% in 2020 compared with 2013, and the industrial water use of the water reception area will increase by 35%^31^. In general, water demand in the water reception area may be 9% higher than in 2013, which is equivalent to 3 billion m^3^ of water. Moreover, 80% of the growth comes from industrial water, of which the reuse of reclaimed wastewater will be the major source^32^.

The result of uncertainty analyses for both the available water and water demand shows that the transferable water in the Danjiangkou reservoir is in line with government planning. Additionally, the water demand in the NCP area will not cause excessive groundwater consumption.

The uncertainty of energy consumption for extraction of groundwater arises mainly due to the water intake, groundwater level, and water pump efficiency. The uncertainty of groundwater reduction has been discussed previously. In this study, future changes in the groundwater table are not considered; however, with the reduction and restriction of groundwater exploitation, the groundwater table will stop dropping or may even pick up. If the groundwater increases or water pump efficiency improves, the energy consumption for groundwater extraction will be further reduced.

## Discussion

With the rapid growth of economic development in the NCP, ensuring water security is an important component of society’s sustainable development. The diverted water from the SNWDP is playing a crucial role in rational water allocation and ecological environment protection. Our new estimation identifies the contribution of the middle route of the SNWDP to energy saving through groundwater replacement. The result shows that in 2030, 6.44 billion m^3^ of groundwater extraction will be replaced by diverted water from the SNWDP, representing 27% of the current groundwater withdrawal. Moreover, as previously mentioned, 2.32 billion kWh energy will be saved, thus, greatly reducing regional energy consumption.

In addition, compared with other water-shortage solutions, reclaimed water consumes 0.4 to 1.0 kWh of energy per ton of water^33^, while seawater desalination consumes 3 to 4 kWh of energy per ton of water^34^. The middle route of the SNWDP could save approximately 0.3 kWh per ton of water, and the project will have non-negligible effects on groundwater protection, energy savings, and carbon dioxide reduction.

## Methods

### Calculation for regional groundwater level and the amount of used groundwater

The groundwater level and groundwater exploitation are affected by the seasonal and inter-annual variation of precipitation. To minimize the annual fluctuation of the groundwater table, we use the average annual groundwater depth from 2004 to 2013. In addition, the data on the regional groundwater level comes from 421 groundwater-level monitoring stations, including 248 national stations in the NCP area^35^ as well as 173 provincial stations from the Hebei Water Resources Department. A dataset of the annual average groundwater depth was created for each observation station using the geographical coordinates. The improved kriging method was used to interpolate the regional groundwater depth with a grid resolution of 1 km × 1 km^36^ in the whole study area. Further, the data on statistical water withdrawal was collected from the annual water resource bulletin published by the provincial and municipal Water Affairs Bureau, including the annual data on water diverted from rivers, pumped water from the subsurface, and consumed water by agriculture and other industries for each irrigation district or county.

### Calculation for energy consumption of groundwater exploitation

Groundwater in North China is well connected and permeable; thus, its exploitation is equivalent to that of underground reservoir water. The energy required to lift groundwater is a function of the gravitational potential energy, and it varies depending on the quantity and depth of the water being pumped and the type and efficiency of the pumping system. This method has been widely used in a number of studies, and the formula is as follows:

1 $$Energy\,consumption(kWh)=\frac{gravity(m{s}^{-2})\times lift(m)\times Mass(kg)}{3.6\times {10}^{6}\times efficiency( \% )},$$

where the gravitational acceleration is 9.8 m/s^2^. The pumping efficiency is mainly affected by the power type, which is mainly electricity or diesel in this study area. In this research, the efficiencies of electrical and diesel motors are estimated to be 65% and 40%^37^, respectively.

By considering the impact of the water table drawdown and water head on the pump lift, the experimental results in 366 villages of North China^38^ show that the pump lift is on average 21.75 m deeper than groundwater level. This relationship was applied to refine the calculation of the pump lift.

2 $$Lift(m)=0.906\times groundwater\,level(m)+21.75\,{R}^{2}=0.62$$

### Calculation of groundwater replacement

We estimate the amount of reduced groundwater exploitation in 2020 and 2030 as the amount of short and long-term groundwater replacement of the SNWDP. After the completion of the SNWDP and its supporting facilities, 9.5 billion m^3^ of water will be delivered to the NCP each year, which will significantly change the water supply structure. After delivery, storage, treatment, and allocation, the diverted water will become one of the main water sources for urban water resources in the near future^17^.

Based on the most stringent water resources management system^39^, the basis for the water resources allocation scheme has been published for the period from 2016 to 2020^20^, and the total volume quota of groundwater exploitation for each year has been established by the provincial, city, and county-level administrators. In addition, the management of groundwater exploitation is mainly focused on urban and industrial areas, and the confined groundwater will prohibit extraction and is reserved only for an urban water emergency. Industrial water will mainly rely on transferred or reclaimed water, while domestic water will make full use of transferred water. However, considering the water safety issue, some groundwater exploitation will remain.

In the 2020 s, groundwater management in rural areas will be gradually put into effect, and part of the groundwater withdrawal will be replaced by SNWDP. This action is targeted to gradually achieve a replenishment balance of the area’s groundwater in 2030. Thus, most of the over-exploited volume of shallow and deep groundwater will be replaced by SNWDP. As shown in Table 1, the amount of reduced groundwater exploitation in 2030 is calculated based on the total volume percentage of groundwater exploitation for each city.

### Analysis of uncertainty in prediction

The amount of groundwater replaced by the SNWDP is mainly affected by two aspects: the water supply capacity of Danjiangkou reservoir and the water demand in the water reception area. Therefore, in this article, the uncertainty analyses focus on the uncertainty of the available water in the Danjiangkou reservoir on the Han River and the uncertainty of water use in the water receiving area.

The uncertainty of the available water in the Danjiangkou reservoir on the Han River is mainly due to the climate change impact on water resources in the upper reaches of the Danjiangkou Reservoir and the water needs throughout the basin.

To analyze the water cycle change trend in the coming years and discuss the water resource variation in the upper reaches of the Danjiangkou reservoir, this study uses the precipitation and runoff projection results of the 21st century for four high-resolution GCMs (Table 2) in the Coupled Model Intercomparison Project Phase 5 (CMIP5) under the RCP 4.5 and RCP 8.5 scenarios. Here, RCP 4.5 represents the mid and stabilizing greenhouse gas emission scenario with climate policies, and RCP 8.5 stands for high greenhouse gas emission in the absence of climate change policies.

To reduce the systematic error in the GCM simulation results, the RCP output from the GCM needs to be corrected for biases^40^. Therefore, a statistical bias correction is applied to monthly precipitation and monthly runoff with the observed East Asia precipitation data^41^ and observed runoff data at the hydrological stations. The correction formulae are as follows:

3 $${P}_{CR}(x,y,t)={P}_{RCP}(x,y,t)\frac{{P}_{obs}(x,y,mon)}{{P}_{GCM}(x,y,mon)\,},$$

4 $${R}_{CR}(x,y,t)={R}_{RCP}(x,y,t)\frac{{R}_{obs}(x,y,mon)}{{R}_{GCM}(x,y,mon)\,}.\,$$

In the formulae, P_RCP_ and R_RCP_ represent the given values of precipitation and runoff to be corrected, respectively, and P_CR_ and R_CR_ represent the corrected values. P_obs_ and R_obs_ are the observed multi-year average precipitation and runoff data during 1962 to 2005, respectively, while P_GCM_ and R_GCM_ are the historical run’s simulation values of the multi-year average precipitation and runoff.

The available water in the Danjiangkou reservoir is calculated by the following formula:

5 $${W}_{RA}=\sum _{i=1}^{n}({W}_{Res}(i)-{W}_{dem}(i)),$$

where ${W}_{RA}$ is the available water of the Danjiangkou reservoir in the i^th^ future climate change scenario, ${W}_{Res}(i)$ is the total water resources in the upper reaches of the Danjiangkou reservoir based on the regional runoff, and ${W}_{dem}(i)$ is the social and economic water demand of the Han River under the i^th^ climate change scenario. The water demand is subdivided into agricultural, industrial, domestic, and environmental water demand. The water demand of agriculture is calculated based on the correlation between precipitation and agricultural water demand from 1990 to 2013. The water demand for industry and domestic use in the Han River is calculated based on the regional future development plan.

The uncertainty of the water demand in the NCP is mainly due to the potential changes in social-ecological water demand and the new water supply project including desalination and recycled water plants. The formula for calculating the water requirement is as follows:

6 $${W}_{NCP}=\sum _{i=1}^{n}{W}_{agr}(i)+{W}_{ind}+{W}_{dom}-{W}_{rec},$$

where, ${W}_{NCP}$ is the water demand of the water reception area in the NCP, and ${W}_{agr}(i)$ is agricultural water demand in the NCP under different climate change.$\,{W}_{ind}$ and ${W}_{dom}$ represent water demand for industry and domestic use in NCP, respectively. ${W}_{rec}$ is the available water from the new water supply project.

### Data Availability

The datasets generated during and/or analysed during the current study are available from the corresponding author on reasonable request.

## References

[1] Liu C, Xia J (2004). Water problems and hydrological research in the Yellow River and the Huai and Hai River basins of China. Hydrol. Process..

[2] Mekonnen MM, Hoekstra AY (2016). Four billion people facing severe water scarcity. Sci. Adv..

[3] Kummu M, Guillaume JHA, Moel HD (2016). The world’s road to water scarcity: shortage and stress in the 20th century and pathways towards sustainability. Sci. Rep..

[4] Feng ZM, Liu DW (2006). A Study on Water Resources Carrying Capacity in Jingjinji Region (in Chinese with English abstract). J. Nat. Resour..

[5] Qiu J (2010). China faces up to groundwater crisi*s*. Nature.

[6] Shi J, Wang Z, Zhang Z (2011). Assessment of deep groundwater over-exploitation in the North China Plain. Geoscience Frontiers.

[7] Liu C, Yu J, Eloise K (2001). Groundwater Exploitation and Its Impact on the Environment in the North China Plain. Water International.

[8] Ministry of Water Resources P.R. China, National Bureau of Statistics P.R. China*. Bulletin of first national census for water* (ChinaWater & Power Press, 2013).

[9] Ministry of Water Resources People’s Republic of China. *China water resources bulletin 2015* (China Waterpower Press, 2016).

[10] Zheng C, Liu J, Cao G (2010). Can China cope with its water crisis?–perspectives from the North China Plain. Groundwater.

[11] Hwang C, Yang Y, Kao R (2016). Time-varying land subsidence detected by radar altimetry: California, Taiwan and north China. Sci. Rep..

[12] Plappally AK, Lienhard VJH (2012). Energy requirements for water production, treatment, end use, reclamation, and disposal. Renew. Sust. Energ. Rev..

[13] Li X, Liu J, Zheng C (2016). Energy for water utilization in China and policy implications for integrated planning. Int. J. Water Resour. D..

[14] Klein, G., Kerbs, M., Hall, V. *et al*. California’s Water – Energy Relationship. http://www.energy.ca.gov/2005publications/CEC-700-2005-011/CEC-700-2005-011-SF.PDF (California Energy Commission, 2005).

[15] Zhang S, Xu L (2007). Energy consumption and the spatial distribution characteristics of shallow groundwater mining in the south Haihe River plain (in Chinese with English abstract). Geographical Research.

[16] Li Y, Xiong W, Zhang W (2015). Life cycle assessment of water supply alternatives in water-receiving areas of the South-to-North Water Diversion Project in China. Water Res..

[17] Liu C, Zheng H (2002). South-to-north Water Transfer Schemes for China. Int. J. Water Resour. D..

[18] Rothausen S, Conway D (2011). Greenhouse-gas emissions from energy use in the water sector. Nat. Clim. Change.

[19] Hu X, Shi L, Zeng J (2016). Estimation of actual irrigation amount and its impact on groundwater depletion: A case study in the Hebei Plain, China. J. Hydrol..

[20] Ministry of Water Resources P.R. China, National Development and Reform Commission, Ministry of Finance P.R. China, The State Council Office of the South to North Water Transfer Project Construction Committee. *The overall reduction use plan of underground water along the middle and east route of the south to north water transfer first-phase project*. (MWR, 2013).

[21] Zhao ZY, Zuo J, Zillante G (2015). Transformation of water resource management: a case study of the South-to-North Water Diversion project. J Clean. Prod..

[22] Beijing Bureau of Statistics, 2013 Beijing national economic and social development statistical bulletin(Beijing Bureau of Statistics, 2014).

[23] Chang C (2010). A multivariate causality test of carbon dioxide emissions, energy consumption and economic growth in China. Appl. Energ..

[24] Barnett J, Rogers S, Webber M (2015). Sustainability: Transfer project cannot meet China’s water needs. Nature.

[25] Thomson A, Calvin K, Smith S (2011). RCP4.5: a pathway for stabilization of radiative forcing by 2100. Climatic Change.

[26] Riahi K, Rao S, Krey V (2011). RCP8.5: a scenario of comparatively high greenhouse gas emissions. Climatic Change.

[27] Zhang X, Xia J (2009). Coupling the hydrological and ecological process to implement the sustainable water resources management in Hanjiang River Basin. Sci. China Technol. Sc..

[28] Zong Y, Yang W, Ma Q (2009). Cassini Growth of Population Between Two MetropolitanCities–A Case Study of Beijing-Tianjin Region, China. Chinese Geogr. Sci..

[29] Ministry of Land and Resources P.R. China, *The national land renovation plan.(2016*–*2020)* (MLR, 2015).

[30] Jiang S, Wang J, Zhao Y (2017). Sustainability of water resources for agriculture considering grain production, trade and consumption in China from 2004 to 2013. J. Clean. Prod..

[31] Ministry of Industry and Information Technology P.R. China, *The green industrial development plan* (*2016–2020*) (MIIT, 2016).

[32] National Development and Reform Commission P.R. China, *The water-saving society construction planning in the 13th Five-year* (NDRC, 2017).

[33] Dreizin Y (2006). Ashkelon seawater desalination project - off-taker’s self costs, 7 supplied water costs, total costs and benefits. Desalination.

[34] Fritzmann C, Lowenberg J, Wintgens T, Melin T (2007). State-of-the-art of reverse osmosis desalination. Desalination.

[35] China GEO-Environmental Monitoring Institute, *China Groundwater Level Yearbook of GEO-Environmental Monitoring* (China Land Press, 2013).

[36] Varouchakis EA, Hristopulos DT, Karatzas GP (2012). Improving kriging of groundwater level data using nonlinear normalizing transformations—a field application. Hydrolog. Sci. J..

[37] Kay, M. *Practical hydraulics* (Spon, 1998).

[38] Wang JX, Rothausen SGSA, Conway D (2012). 2012 China’s water energy nexus: greenhouse-gas emissions from groundwater use for agriculture. Environ. Res. Lett..

[39] The State Council of the People’s Republic of China, the water supply management regulation of the South to North Water Diversion Project (The State Council, 2014).

[40] Arnell N (2004). Climate change and global water resources: SRES emissions and socio-economic scenarios. Global Environ. Change.

[41] Xie P, Yatagai A, Chen M (2007). A gauge-based analysis of daily precipitation over East Asia. J. Hydrometeorol..

[42] Chylek P, Li J, Dubey M (2011). Observed and model simulated 20th century Arctic temperature variability: Canadian Earth System Model CanESM2. Atmos. Chem. Phys..

[43] Voldoire A, Sanchez-Gomez E, Mélia D (2013). The CNRM-CM5. 1 global climate model: description and basic evaluation. Clim. Dynam..

[44] Nazarenko L, Schmidt G, Miller R (2015). Future climate change under RCP emission scenarios with GISS ModelE2. J. Adv. Model. Earth Sy..

[45] Yukimoto S, Adachi Y, Hosaka M (2012). A New Global Climate Model of the Meteorological Research Institute: MRI-CGCM3 —Model Description and Basic Performance. J. Meteorol. Soc. Jpn..

